# Impact of Comorbidity Burden on Clinical Outcomes in Older Adults With Metastatic Colorectal Cancer: A Systematic Review and Meta-Analysis

**DOI:** 10.7759/cureus.94099

**Published:** 2025-10-08

**Authors:** Ryuichi Ohta, Yudai Tanaka, Kaoru Tanaka, Hidetoshi Hayashi

**Affiliations:** 1 Community Care, Unnan City Hospital, Unnan, JPN; 2 Commnity Care, Unnan City Hospital, Unnan, JPN; 3 Medical Oncology, Kindai University Faculty of Medicine, Sayama, JPN

**Keywords:** adverse event, aged, charlson comorbidity index, colorectal neoplasms, mortality, multimorbidity, neoplasm metastasis, prognosis, progression free survival

## Abstract

Older adults represent most patients with metastatic colorectal cancer (mCRC), yet their management is often complicated by multimorbidity. Comorbid conditions may influence treatment selection, tolerance, and survival, but the prognostic role of comorbidity burden in mCRC remains unclear. We conducted a systematic review and meta-analysis to assess the association between comorbidity, measured by the Charlson Comorbidity Index (CCI), and clinical outcomes in older patients with mCRC. Following the Preferred Reporting Items for Systematic Reviews and Meta-Analyses (PRISMA) 2020 guidelines, we systematically searched PubMed, Embase, and Web of Science (2000 to April 2025) for studies of adults aged ≥65 years with mCRC that assessed clinical outcomes according to baseline comorbidity, as measured by the CCI. Eligible endpoints included overall survival (OS), progression-free survival (PFS), and treatment-related adverse events (AEs). Data were extracted in duplicate, and study quality was appraised using the Newcastle-Ottawa Scale. Random-effects models were applied to pool hazard ratios (HRs). Fourteen studies involving 16,736 patients met the inclusion criteria. Thirteen studies reported OS, two reported PFS, and two reported AEs. Higher comorbidity burden was significantly associated with worse OS (pooled HR = 1.19, 95% CI: 1.02-1.39; I² = 84.1%). No significant difference was observed for PFS (pooled HR = 1.00, 95% CI: 0.89-1.14; I² = 4.9%). For AEs, estimates were imprecise, with wide confidence intervals suggesting uncertainty about the association between high CCI and increased risk (pooled HR = 1.73, 95% CI: 0.79-3.79; I² = 86.4%). Multimorbidity, as measured by the CCI, is modestly associated with poorer overall survival in older mCRC patients (HR ≈ 1.19), but does not appear to influence progression-free survival or treatment-related toxicity consistently. Given the substantial heterogeneity across studies and the limited data on progression-free survival and adverse events, these findings should be interpreted with caution. Nevertheless, they suggest that comorbidity should guide, but not preclude, standard therapy, underscoring the importance of individualized, non-ageist treatment strategies.

## Introduction and background

Metastatic colorectal cancer (mCRC) is a significant public health concern worldwide, including in Japan [1]. The incidence of colorectal cancer increases with age, with patients aged 70 years or older comprising the majority of new diagnoses [1]. Once distant metastases are present, curative treatment is rarely feasible, and management requires a multidisciplinary approach, including chemotherapy and palliative care [2]. However, elderly patients often experience barriers to receiving standard therapy due to physiological decline, functional heterogeneity, and comorbid conditions, making individualized treatment strategies essential [3].

Among these factors, multimorbidity, which is the coexistence of two or more chronic conditions, is critical in the treatment of older patients with mCRC. Common comorbidities include cardiovascular disease, diabetes mellitus, chronic kidney disease, chronic obstructive pulmonary disease (COPD), and dementia [4]. These conditions can influence eligibility for anticancer therapy, treatment intensity, risk of adverse events (AEs), treatment discontinuation, and ultimately prognosis [5]. Elderly patients with mCRC often face challenges related not only to age but also to multiple coexisting health conditions. Several studies suggest that comorbidity burden may exert a greater influence on clinical outcomes than chronological age alone [6]. This highlights the importance of systematically evaluating the role of comorbidity, particularly as quantified by the Charlson Comorbidity Index (CCI), to inform treatment decisions better and improve prognostic accuracy in this population.

In oncology research, multimorbidity has been operationalized using several approaches, ranging from comprehensive geriatric assessment (CGA) tools to simpler indices such as the Cumulative Illness Rating Scale [7,8]. The CCI, however, remains the most widely applied measure in large-scale clinical and epidemiological studies because of its practicality and validated prognostic value across cancer populations [7,8]. For this reason, we focused on the CCI as the primary metric of comorbidity in the present review. Existing evidence is fragmented, heterogeneous in methodology, and often underpowered, leaving uncertainty regarding the actual prognostic impact of comorbidities [7,8]. For example, studies vary in design (retrospective vs prospective cohorts) and in how comorbidity is categorized, with some applying different cutoffs or score groupings for the CCI. Thus, a systematic review is warranted to clarify these associations and provide evidence to guide clinical practice.

This systematic review aims to synthesize the available evidence on the relationship between comorbidity burden, primarily assessed by the CCI, and outcomes in older patients with mCRC. Specifically, this review examined associations with overall survival (OS), progression-free survival (PFS), and AEs. By consolidating current knowledge, the review aimed to enhance prognostic prediction, inform individualized treatment strategies, and support updates to clinical guidelines for elderly patients with mCRC.

## Review

Methods

Study Design

This study was conducted as a systematic review in accordance with the Preferred Reporting Items for Systematic Reviews and Meta-Analyses (PRISMA) 2020 guidelines [9]. The protocol was prospectively registered in PROSPERO to ensure transparency and reproducibility (ID: CRD420251147839).

Data Sources and Search Strategy

We systematically searched PubMed, Embase, and Web of Science for eligible studies. Additional sources, such as the Cochrane Library and Scopus, were considered relevant. The search period was restricted from January 2000 to April 2025 to reflect contemporary advances in diagnosis and treatment. Only articles published in English were included; Japanese-language studies were considered on a case-by-case basis to ensure consistency of data extraction. Two bilingual investigators reviewed these studies, and relevant data were translated into English before being cross-checked against the extraction template to maintain reproducibility. The following keywords and Medical Subject Headings (MeSH) were applied: (“Colorectal Neoplasms” OR “colorectal cancer” OR “colon cancer” OR “rectal cancer”) AND (“Neoplasm Metastasis” OR “metastatic” OR “stage IV”) AND (“Aged” OR “elderly” OR “older adult” OR “65 years”) AND (“Multimorbidity” OR “Comorbidity” OR “Charlson Comorbidity Index” OR “CCI”) AND (“Survival” OR “Prognosis” OR “Treatment Outcome” OR “chemotherapy” OR “overall survival” OR “treatment completion”). All search strategies, including search terms, dates, and the number of records retrieved, were documented for reproducibility.

Eligibility Criteria

Studies were considered eligible if they investigated older adults (defined as those aged 65 years or older) diagnosed with mCRC and evaluated multimorbidity using the CCI. When studies applied alternative age thresholds (e.g., ≥70 years), they were included if the population was clearly described as elderly and otherwise met the eligibility criteria. Eligible studies were required to report OS, PFS, or AEs. Both prospective and retrospective observational designs, as well as post hoc analyses of clinical trials that presented stratified results according to comorbidity burden, were included.

Studies were excluded if they focused exclusively on patients without metastatic disease, did not utilize the CCI, or enrolled only pediatric or younger adult populations. Non-original studies such as narrative reviews, systematic reviews, editorials, and conference abstracts were excluded, as were single-case reports. In addition, studies that did not explicitly analyze the relationship between comorbidity and clinical outcomes of interest were deemed ineligible.

Study Selection

Two reviewers independently screened titles and abstracts. Full texts of potentially eligible studies were then reviewed to determine final inclusion. Disagreements were resolved through discussion, and if necessary, a third reviewer adjudicated.

Data Extraction

Data extraction was performed using a standardized form in Covidence (Veritas Health Innovation, Melbourne, Australia). Two reviewers independently extracted data on study characteristics, patient demographics, comorbidity assessment, and outcomes, with discrepancies resolved through discussion or consultation with a third reviewer. Extracted items included: study characteristics (author, year, country, and study design), patient characteristics (age, sex, and sample size), tumor features (primary site (right vs. left), RAS/RAF mutation status, and microsatellite instability (MSI) status), CCI score, treatment details (chemotherapy regimens, and supportive/palliative interventions), clinical outcomes (OS and PFS) and follow-up duration and attrition. Quality assessment of included observational studies was conducted using the Newcastle-Ottawa Scale (NOS) [10]. Sensitivity analyses were planned to exclude studies with low NOS scores. In addition, exploratory subgroup analyses were considered according to NOS domains (e.g., selection, comparability, and outcome) to examine whether specific sources of bias influenced the overall findings.

Data Synthesis

Where feasible, effect sizes (hazard ratios, risk ratios, odds ratios) were extracted and pooled using a random-effects model (DerSimonian and Laird method). Heterogeneity was assessed with the I² statistic, with I² ≥50% indicating substantial heterogeneity. Meta-regression analyses were planned to explore potential sources of heterogeneity, including study-level covariates such as age threshold (≥65 vs ≥70 years), study region, and treatment type. Publication bias was assessed using funnel plots and Egger’s test, but only when at least 10 studies were available, in line with methodological recommendations. All statistical analyses were conducted in Python (version 3.11) using the pandas, NumPy, SciPy, and statsmodels packages [11].

Results

Study Selection

The initial search of three electronic databases (Embase, PubMed, and Web of Science) yielded 1,097 records, including 777 from Embase, 210 from PubMed, and 110 from Web of Science. After removal of 199 duplicates (198 identified automatically by Covidence and one identified manually), 898 unique records remained for screening. Titles and abstracts were screened by two independent reviewers, resulting in the exclusion of 799 records that did not meet the eligibility criteria.

**Figure 1:**
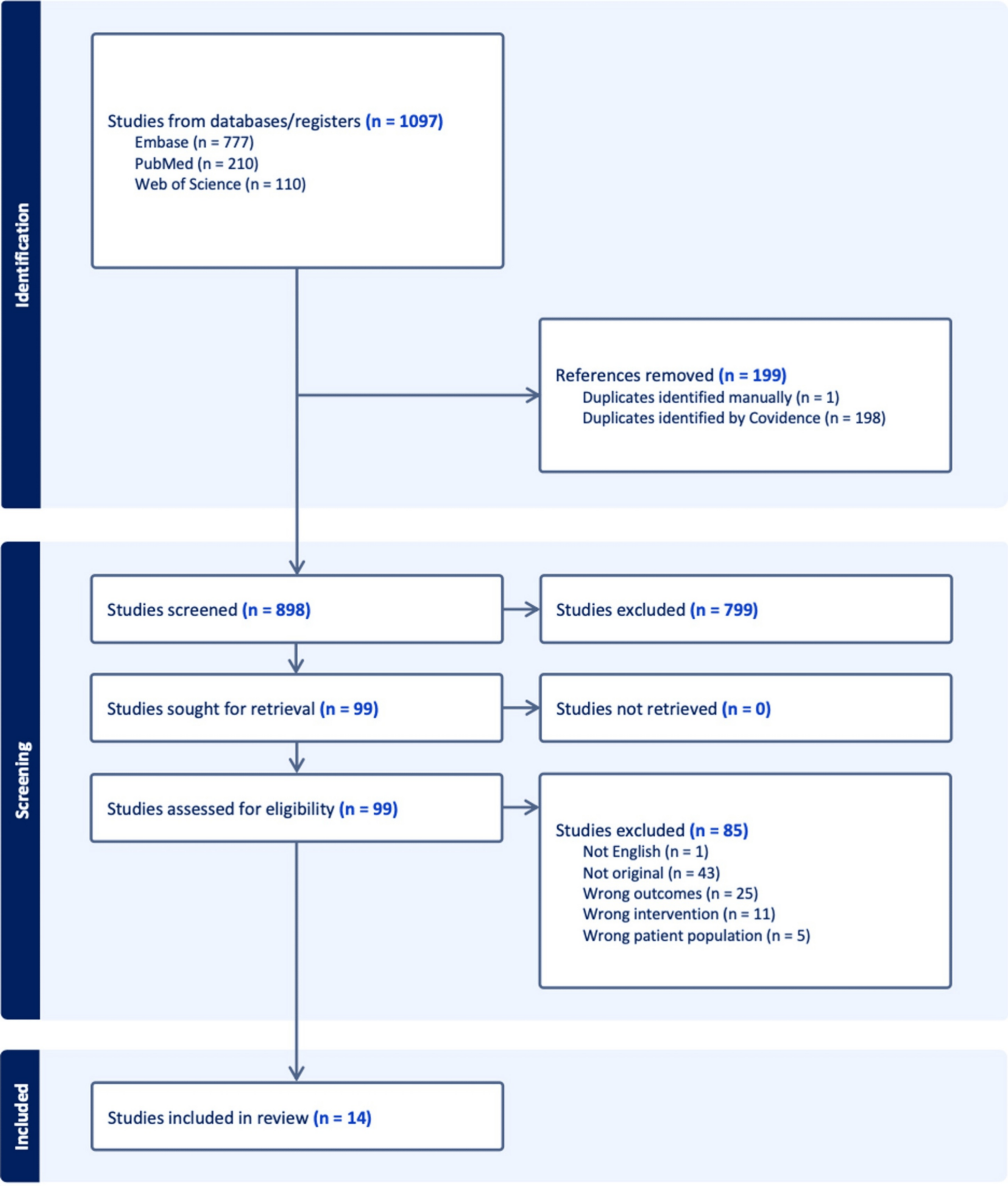
Selection flow.

Ninety-nine articles were retrieved in full text for detailed evaluation. Of these, 84 studies were excluded for the following reasons: non-English language publication (n = 1), non-original studies such as reviews or editorials (n = 43), ineligible intervention or exposure (n = 11), ineligible patient population (n = 5), or outcomes not relevant to the review (n = 25). Ultimately, 14 studies met the inclusion criteria and were included in the systematic review. The study selection process is summarized in a PRISMA 2020 flow diagram. The detailed process of study identification, screening, and inclusion is illustrated in the PRISMA flow diagram (Figure 1).

The Characteristics of the Included Studies

A total of 14 studies met the eligibility criteria, of which 13 reported OS, two reported PFS, and two reported treatment-related AEs. Publications spanned the years 2007-2022 and were conducted across multiple regions, most commonly in the United States (n = 3), Australia (n = 3), and Germany (n = 2), with additional single-country cohorts from other locations. Across all included reports, the cumulative study population comprised approximately 16,736 older adults with mCRC. Individual study sample sizes ranged from 95 to 2,726 participants (median, 1,085).

Across the 14 included studies, the median or mean age consistently reflected an elderly mCRC population. Most cohorts defined the elderly as individuals aged 65 years or older, with some reporting further stratification into older age bands (e.g., 75-84 years or ≥85 years). The overall median ages ranged from the mid-60s to mid-80s, demonstrating that the included studies covered a broad spectrum of older adults, including the very elderly.

Sex distribution was variably reported but showed a predominance of male patients in most cohorts. Male proportions generally ranged between 55% and 65%, although there were notable outliers: Wang et al. (2007) and Badic et al. (2022) reported nearly equal or female-predominant populations (46% and 44% male, respectively), while Gonsalves et al. (2012) reported an exceptionally high proportion of male patients (98%). Only a few studies therefore enrolled more women than men.

Definitions of “elderly” varied: ≥65 years in five studies and ≥70 years in seven studies. Comorbidity was assessed using Charlson-based measures in all reports (Charlson/CCI or equivalent), with age-adjusted CCI explicitly stated in four studies. High versus low comorbidity was most frequently dichotomized at CCI ≥3 (8/14 studies); two studies used ≥2, one treated CCI as a continuous variable, and three adopted multilevel strata (e.g., 0/1/2/≥3 or broader bands).

Regarding disease and treatment context, most cohorts enrolled patients with colon and/or rectal primaries, with colon involvement cited more often than rectal disease in study descriptions. Where metastatic patterns were specified, liver involvement was the most commonly reported site, followed by lung and peritoneum; several cohorts distinguished synchronous from metachronous presentation. Performance status reporting was heterogeneous, but narratives frequently indicated a predominance of ECOG 0-1, with a non-negligible share of ECOG ≥2 in real-world cohorts.

First-line systemic therapy typically included FOLFOX or FOLFIRI (with fluoropyrimidine monotherapy in select contexts), and targeted agents were frequently used: bevacizumab was mentioned in most studies, and anti-EGFR therapy (cetuximab or panitumumab) in roughly half. Several reports also discussed local interventions in selected patients (e.g., hepatic resection, stenting, or radiotherapy). Where treatment uptake metrics were available, initiation rates were generally high and completion rates lower, consistent with the competing risks and tolerability challenges in older, comorbid populations (Table 1).

**Table 1:** Characteristics of the included articles. Age is reported as the median or mean with range where available. Sex distribution is presented as the proportion of male and female patients as reported in the original publications. Reported outcomes indicate whether the study provided hazard ratios for OS, PFS, and/or treatment-related AEs stratified by the CCI or its variants. OS, overall survival; PFS, progression-free survival; AEs, adverse events; CCI, Charlson Comorbidity Index; CLM, colorectal liver metastases; TRACC, Treatment of Recurrent and Advanced Colorectal Cancer registry; CALGB, Cancer and Leukemia Group B; SWOG, Southwest Oncology Group; PSM, propensity score matching; PS, performance status; CRC, colorectal cancer; MWA, microwave ablation.

Study	Study design	Sample size (N)	Age (median/mean, range)	Sex distribution (%)	Reported outcome
Wang et al., 2007 [12]	Population-based retrospective cohort study	N = 923; metachronous CLM: 514; synchronous CLM: 409	All ≥65 years; distribution: 65–69 (32%), 70–74 (35%), 75–79 (20%), ≥80 (12%)	Female 54%, male 46%	OS
Gonsalves et al., 2012 [13]	Retrospective, population-based cohort	2,625 patients with stage IV colon cancer undergoing resection	<65: 41%; 65–79: 47%; ≥80: 11%	Male 98%, female 2%	OS
Field et al., 2014 [14]	Prospective, multicenter registry analysis	1065 patients (private 558, public 507)	Median 68.3 years (range 18–97); ≤70: 52% private vs 56% public; >70: 48% vs 44%	Male 59%, female 41%	OS
Jehn et al., 2014 [15]	Non-interventional study (NIS), retrospective subgroup analysis	497 patients in total; <65 years: 247, ≥65 years: 250	Overall median: 66; <65 group: 59 (30–65), ≥65 group: 70 (66–88)	Male: 64%, female: 36% (similar among age groups)	PFS, AE
Parakh et al., 2015 [16]	Secondary analysis of prospective registry data (TRACC registry)	821 elderly patients (≥65 years): 65–74 years (n = 363), 75–84 years (n = 352), ≥85 years (n = 106)	Overall median: 76; 70 (65–74 years), 80 (75–84 years), 87 (≥85 years)	Male: 59%, female: 41%; female proportion increased with age (35%, 43%, 54%)	OS
Ahmed et al., 2017 [17]	Population-based cohort (registry)	1947 patients	Median 70 (IQR 60–78)	M:F ≈ 1.3:1	OS
Seidensticker et al., 2018 [18]	Retrospective single-institution cohort	266 patients	Mean: 63 years (SD 9.7), mean at first intervention: 66.5 years (SD 9.6); ≥70 years = 89 (33.5%)	Male: 67.3%, Female: 32.7%	OS
Seoane et al., 2020 [19]	Retrospective observational cohort (2005–2012, two centers)	N = 95 (Stent n = 46, Surgery n = 49)	Mean: 67 ± 11.9 years (stent 68.9, surgery 65.2, p = 0.1)	60% male, 40% female (similar across groups)	OS
Tinguely et al., 2020 [20]	Retrospective, nationwide registry (SweLiv) with propensity score matching	727 in total (MWA 82, resection 645); after PSM: MWA 70, resection 201	Median: MWA 70 (range 28–86), resection 68 (24–88); after PSM: both 69 years	Male/female: MWA 46/36, resection 403/242; after PSM ~50/50	OS
Travers et al., 2020 [21]	Retrospective analysis of prospective registry (TRACC registry)	2726 (good PS 2215, poor PS 329, very poor PS 182)	Median: good PS 65 years, poor PS 76 years, very poor PS 79 years (range 18–100)	Male 60% (good), 56% (poor), 50% (very poor)	OS
Niedersüß-Beke et al., 2021 [22]	Retrospective, multicenter (registry + chart review)	1105 patients in total; CCI/aaCCI available for 577 patients	Median 69 (IQR 60–76)	Female 37.4%, male 62.6%	OS
Kellokumpu et al., 2021 [23]	Retrospective, population-based cohort	1479 CRC patients (2000–2015)	Mean: colon 70.7 (SD 11.3), rectum 67.9 (SD 10.7). Low ACCI = younger, high ACCI = older	Colon: male 48.7%; rectum: male 64.8%	OS
Badic et al., 2022 [24]	Retrospective, population-based cohort	1115 patients	Median 85 (range 80–103)	Male 44%, female 56%	OS
McCleary et al., 2022 [25]	Secondary analysis of a prospective randomized phase III trial (CALGB/SWOG 80405)	1345 (755 <70 years with no comorbidity, 340 <70 years with comorbidity, 128 ≥70 years with no comorbidity, 122 ≥70 years with comorbidity)	Median 59 years (IQR 51–68); <70 years = 81.4% (n=1095), ≥70 years = 18.6% (n=250); subgroup ≥70: 70–74 years = 57.6%, 75–79 years = 32.8%, 80–84 years = 8.8%, 85+ = 0.8%	Male 59.0%, female 41.0%	OS, PFS, AE

Overall Survival

Thirteen studies reporting OS were included in the quantitative synthesis. When pooled using a random-effects model, higher CCI scores were significantly associated with worse OS compared with lower CCI scores (pooled hazard ratio (HR) = 1.19, 95% confidence interval (CI): 1.02-1.39; k = 13). Although statistically significant, this represents only a modest increase in relative risk. In practical terms, this effect suggests that higher comorbidity may result in a slight but meaningful decrement in survival, underscoring the importance of considering comorbidity in prognostic assessments without overestimating its impact. Considerable statistical heterogeneity was observed (Q = 75.5, df = 12, p < 0.001; I² = 84.1%), indicating substantial variability across studies. Possible sources of heterogeneity include differences in treatment era (with earlier cohorts receiving less intensive systemic therapy), geographic region (population-based registry studies from North America and Europe vs. single-institution series), and baseline demographics (median age varying from the mid-60s to mid-80s). In addition, definitions of high versus low comorbidity varied across studies, with some using binary cut-offs based on the CCI, while others applied age-adjusted scores. Although subgroup analyses were limited by statistical power, these contextual differences likely contributed to the observed inconsistency in effect estimates.

**Figure 2:**
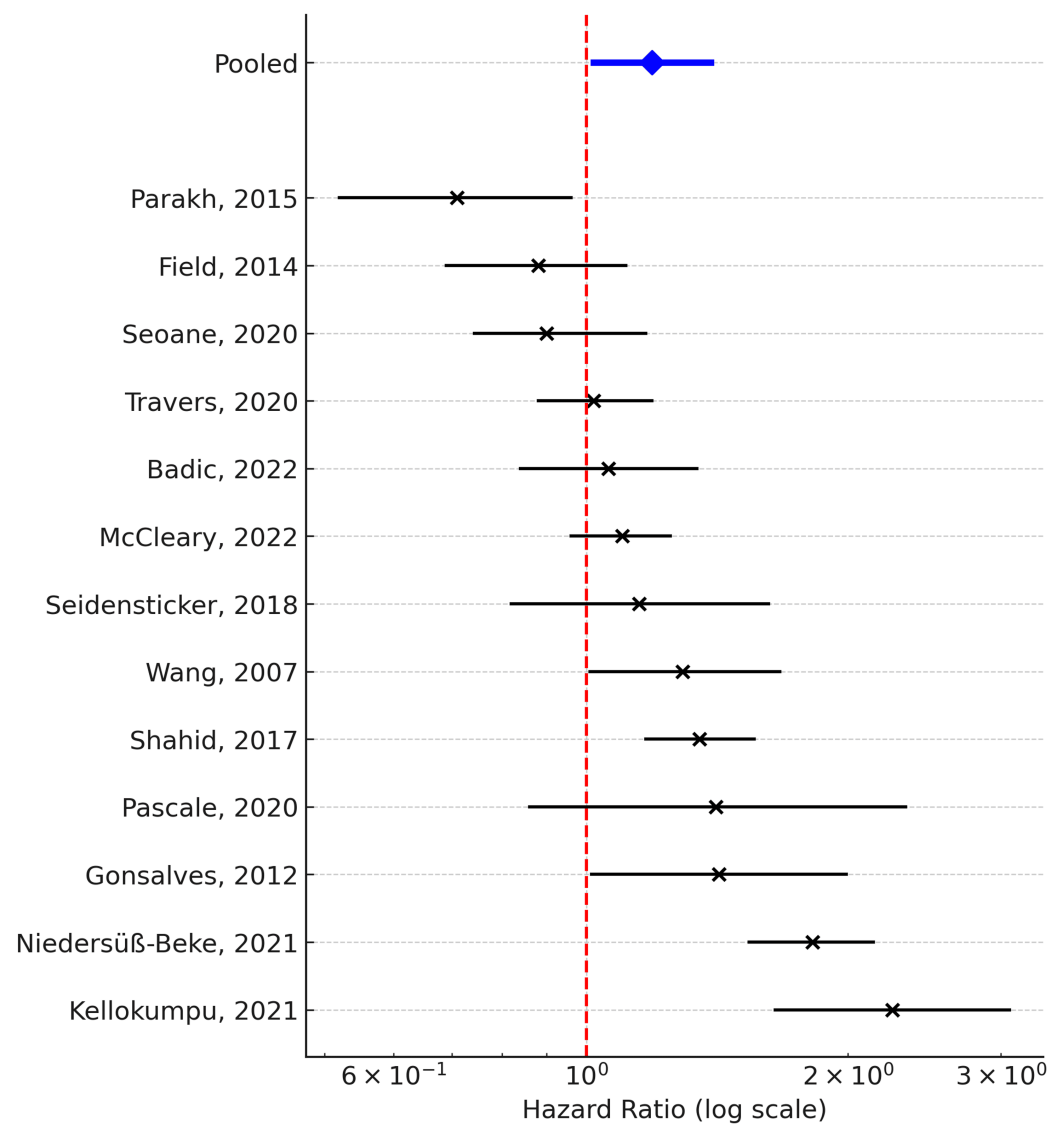
Forest plot of overall survival (OS) comparing high versus low Charlson Comorbidity Index (CCI) groups among elderly patients with metastatic colorectal cancer. Hazard ratios (HRs) with 95% confidence intervals (CIs) from 13 studies are presented. The pooled effect estimate is shown as a blue diamond (pooled HR = 1.19, 95% CI: 1.02–1.39) [11-13,15-24]. The vertical dashed line represents the null effect (HR = 1).

The corresponding forest plot illustrates that, although effect estimates varied, the majority of included studies demonstrated a consistent trend toward poorer survival in patients with higher comorbidity burden. Subgroup analyses stratified by CCI cut-off values (e.g., 0-2 vs. ≥3) were attempted; however, the number of studies using harmonized thresholds was insufficient to yield reliable pooled estimates (Figure 2).

Progression-Free Survival

Two studies reported PFS and were included in the quantitative synthesis. The pooled analysis demonstrated no significant difference in progression risk between patients with higher versus lower CCI scores (pooled HR = 1.00, 95% CI: 0.89-1.14; k = 2). Statistical heterogeneity was low (Q = 1.05, df = 1, p = 0.31; I² = 4.9%), indicating consistent results across the limited available studies.

**Figure 3:**
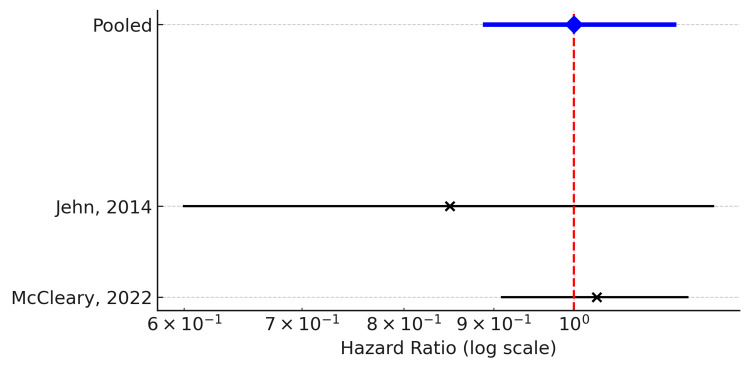
Forest plot of progression-free survival (PFS) comparing high versus low Charlson Comorbidity Index (CCI) groups among elderly patients with metastatic colorectal cancer. Hazard ratios (HRs) with 95% confidence intervals (CIs) from 2 studies are shown. The pooled effect estimate (blue diamond) indicated no significant association between CCI and PFS (pooled HR = 1.00, 95% CI: 0.89–1.14) [14,24]. The vertical dashed line represents the null effect (HR = 1).

The forest plot illustrates that both studies individually suggested comparable PFS outcomes regardless of comorbidity burden. Given the small number of eligible studies, subgroup or sensitivity analyses were not feasible (Figure 3).

Adverse Events

Two studies evaluated the impact of the CCI on treatment-related AEs. The pooled analysis demonstrated a trend toward higher risk of AEs in patients with higher CCI scores; however, the association did not reach statistical significance (pooled HR = 1.73, 95% CI: 0.79-3.79; k = 2). Considerable heterogeneity was observed (Q = 7.34, df = 1, p = 0.0068; I² = 86.4%), indicating substantial variability between the two studies in terms of patient populations, treatment regimens, and definitions of AEs.

**Figure 4:**
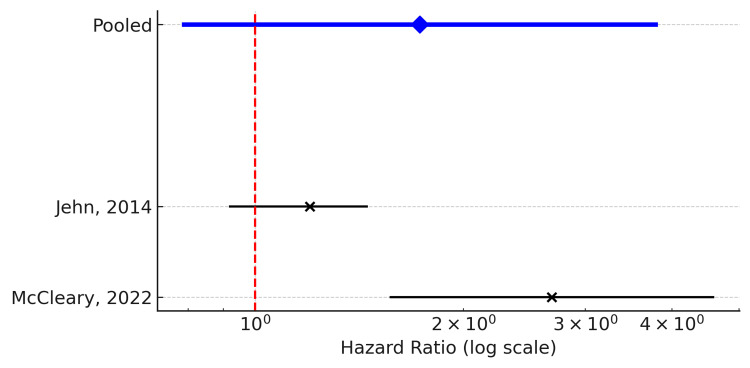
Forest plot of treatment-related adverse events (AEs) comparing high versus low Charlson Comorbidity Index (CCI) groups among elderly patients with metastatic colorectal cancer. Hazard ratios (HRs) with 95% confidence intervals (CIs) from 2 studies are shown. The pooled effect estimate (blue diamond) suggested a non-significant trend toward higher AE risk in high-CCI patients (pooled HR = 1.73, 95% CI: 0.79–3.79) [14,24]. The vertical dashed line indicates the null effect (HR = 1).

The forest plot illustrates these divergent results, with one study suggesting a pronounced increase in AEs among high-CCI patients. At the same time, the other reported a more modest or nonsignificant difference. Given the limited number of studies, further subgroup or sensitivity analyses were not feasible (Figure 4).

Publication Bias Assessment

**Figure 5:**
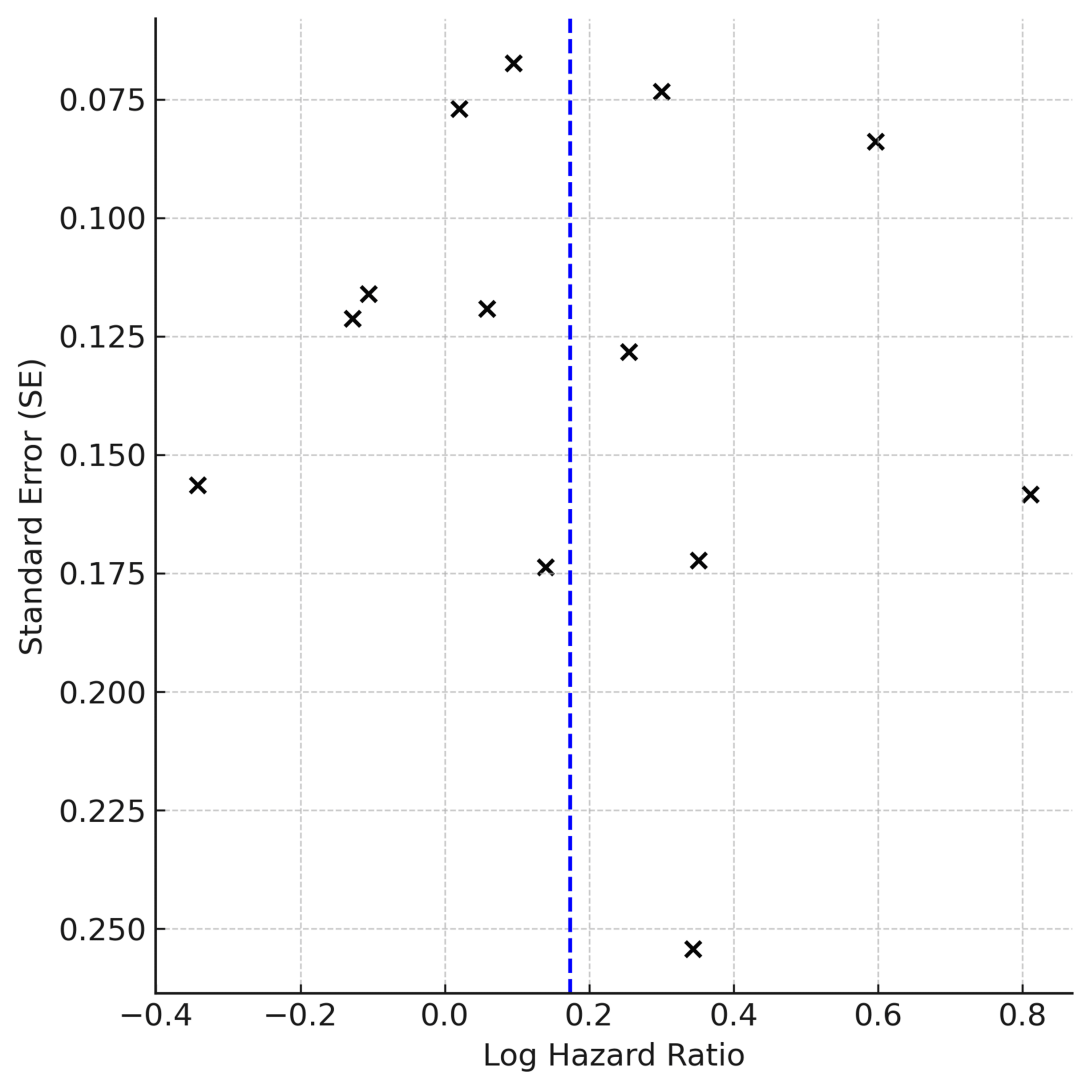
Funnel plot assessing publication bias for overall survival (OS) across 13 studies. Each dot represents an individual study, plotting the log hazard ratio against its standard error (SE) [11-13,15-24]. The vertical dashed line indicates the pooled effect estimate (log HR corresponding to HR = 1.19). Visual asymmetry suggests potential small-study effects; however, Egger’s test did not show statistically significant publication bias.

Publication bias was formally assessed only for OS, as this outcome included more than ten studies (k = 13). Visual inspection of the funnel plot suggested a degree of asymmetry, with smaller studies tending to report larger hazard ratios, raising the possibility of publication bias or small-study effects. However, Egger’s regression test did not reach statistical significance (p > 0.05), indicating that the observed asymmetry may also reflect underlying clinical and methodological heterogeneity rather than selective reporting. For PFS (k = 2) and AEs (k = 2), the number of studies was insufficient to conduct reliable assessments of publication bias; therefore, no funnel plots or formal statistical tests were performed (Figure 5).

Sensitivity Analyses and Subgroup Analyses

Exploratory subgroup analyses were conducted to examine the robustness of the findings for OS. Several studies applied different thresholds to define high versus low CCI groups (e.g., 0-2 vs. ≥3, or dichotomization at the median value). However, the number of studies using harmonized cut-offs was insufficient to permit reliable pooled estimates within subgroups. Consequently, results should be interpreted with caution, as variability in CCI categorization may have contributed to the observed heterogeneity.

Sensitivity analyses excluding individual studies one at a time (leave-one-out analysis) did not materially alter the overall pooled estimate for OS, indicating that no single study disproportionately influenced the results. For PFS and AEs, the limited number of eligible studies (k = 2 each) precluded meaningful sensitivity or subgroup analyses.

Quality Assessment

The methodological quality of the included studies was assessed using the NOS [10]. Overall, most studies were of moderate to high quality, with total scores ranging from 4 to 8 out of 9. The main limitations were inadequate adjustment for confounding and incomplete outcome ascertainment (Table 2).

**Table 2:** Quality assessment of included studies using NOS. Quality assessment of included studies was conducted using the Newcastle–Ottawa Scale (NOS) for cohort studies. The NOS assigns up to 4 points for Selection, 2 points for Comparability, and 3 points for Outcome, with a maximum total score of 9. Higher scores indicate better methodological quality.

First author (Year)	Selection (0–4)	Comparability (0–2)	Outcome (0–3)	Total (0–9)
Wang (2007) [12]	3	1	2	6
Gonsalves (2012) [13]	4	2	2	8
Field (2014) [14]	3	2	2	7
Jehn (2014) [15]	3	1	1	5
Parakh (2015) [16]	4	2	2	8
Ahmed (2017) [17]	3	2	2	7
Seidensticker (2018) [18]	3	1	2	6
Seoane (2020) [19]	2	1	1	4
Tinguely (2020) [20]	4	2	2	8
Travers (2020) [21]	3	2	2	7
Niedersüß-Beke (2021) [22]	3	2	2	7
Kellokumpu (2021) [23]	4	2	2	8
Badic (2022) [24]	3	2	2	7
McCleary (2022) [25]	3	2	2	7

Discussion

Summary of the Study

This systematic review and meta-analysis demonstrate that a higher comorbidity burden, operationalized primarily by the CCI, is associated with modestly worse OS among older adults with mCRC (pooled HR 1.19, 95% CI 1.02-1.39). In contrast, pooled analyses found no clear association between comorbidity burden and PFS (pooled HR 1.00, 95% CI 0.89-1.14) and only a nonsignificant trend toward more treatment-related AEs in high-CCI groups (pooled HR 1.73, 95% CI 0.79-3.79). Considerable heterogeneity (I² = 84% for OS) likely reflects differences in CCI thresholds, treatment eras and strategies, and patient selection across studies. Taken together, these findings suggest that while comorbidity contributes to inferior long-term survival, likely through noncancer mortality, treatment selection, and early discontinuation, it should not automatically preclude offering standard systemic therapy at appropriate intensity. Beyond these clinical mechanisms, biological plausibility also exists: multimorbid patients may experience chronic inflammation, immune senescence, and impaired physiological reserve, which could adversely influence both cancer progression and treatment tolerance. Therefore, therapeutic decisions should avoid ageism and instead be guided by a structured assessment of comorbidity alongside functional status and patient goals.

Comparison With Other Studies

Our results align with prior work showing that multimorbidity adversely influences survival among patients with advanced cancers, including CRC, and that its prognostic effect is at least as salient as chronological age alone [26,27]. The absence of a clear PFS signal in our meta-analysis echoes observations from contemporary registries and post hoc trial analyses in which comorbidity does not consistently predict tumor-centric endpoints once patients initiate therapy. This pattern supports the hypothesis that the survival decrement associated with comorbidity is driven by factors beyond intrinsic tumor biology, such as competing noncancer mortality, reduced treatment receipt or dose intensity, interruptions from decompensated comorbid conditions, and heightened vulnerability to intercurrent illness, rather than accelerated disease progression per se [28,29]. Similarly, the nonsignificant but heterogeneous AE signal is consistent with mixed reports in the literature: some cohorts show more toxicity in multimorbid patients, whereas others demonstrate comparable tolerability when regimens are selected and monitored judiciously [15,25]. Collectively, the literature and our synthesis support a nuanced approach: comorbidity should calibrate, not curtail, treatment.

Strengths of the Study

This review has several notable strengths. By focusing specifically on older adults with mCRC, it addresses an underrepresented yet clinically important population. Synthesizing results from large real-world cohorts and a phase III trial enhances both relevance and generalizability. Outcomes of interest, i.e., OS, PFS, and AEs, were prespecified, and adjusted hazard ratios were extracted where possible, ensuring analytical rigor. The consistent use of the CCI, a validated and transportable tool, provides a practical benchmark for clinicians and future research. Importantly, the finding of worse OS without parallel differences in PFS or AEs highlights that multimorbidity influences prognosis largely through competing risks and treatment allocation, underscoring the importance of careful, non-ageist treatment strategies [30-32].

Limitations

This study has several limitations. Variability in CCI ascertainment (classic vs age-adjusted), cut points (for example, ≥3 vs ≥2 vs continuous), and case mix (metastatic distribution, synchronous vs metachronous, ECOG status) contributed to high I² for OS, limiting the interpretability of subgroup analyses. The included studies spanned chemotherapy-only eras to targeted-therapy periods with evolving supportive care; unmeasured secular trends likely affected the outcomes. Most evidence is observational. Treatment allocation, dose intensity, and early discontinuation are influenced by comorbidities and performance status, potentially leading to confounding by indication despite multivariable adjustment. Competing-risk dynamics (noncancer death) were seldom explicitly modeled. Only two studies informed PFS and AEs each, precluding robust meta-regression, formal exploration of dose intensity, hospitalization, or treatment completion, and limiting precision around safety estimates. The CCI does not capture the severity of multimorbidity, frailty, cognition, nutrition, polypharmacy, or social support. This underscores the added value of CGA, which systematically evaluates these domains and may refine prognostication and guide individualized treatment planning beyond what the CCI alone can provide. Similarly, frailty indices and claims-based severity weights could complement the CCI in future research. In addition, inconsistent reporting of molecular features (RAS/RAF, MSI), local therapies, and supportive interventions limited the ability to adjust for disease biology and the effects of multidisciplinary care. Publication bias could not be excluded, although Egger’s test for OS was not significant; small-study effects may persist. Although multinational, many cohorts were from high-income settings; the applicability to other health systems and to very old populations (≥85 years) or those with high frailty remains uncertain.

## Conclusions

Among older adults with mCRC, greater comorbidity burden is associated with modestly worse OS but not clearly with shorter PFS or higher treatment-related toxicity in pooled estimates. These findings argue against reflexive de-escalation or denial of active therapy based solely on comorbidity counts. Instead, clinicians should (1) perform structured assessments that combine the CCI with functional status and geriatric domains, (2) optimize comorbid conditions and supportive care before treatment, and (3) individualize regimen choice and dosing with close toxicity monitoring. Future research should prioritize prospective cohorts and pragmatic trials that stratify or randomize by standardized comorbidity and frailty measures, report harmonized CCI thresholds, model competing risks, and incorporate patient-centered endpoints such as treatment completion, dose intensity, hospitalizations, and quality of life. Such work will help translate the observed OS signal into actionable care pathways that preserve efficacy while minimizing harm for multimorbid older adults with mCRC.
